# Salvage sequential integrated boost radiotherapy followed by sintilimab–bevacizumab in cervical small-cell carcinoma with >10 brain metastases: A 3-year survivor case report

**DOI:** 10.3389/fimmu.2025.1590848

**Published:** 2025-10-09

**Authors:** Yuanyuan Xu, Xiang Gong, Huailin He, Xiangyu Deng, Zhenhua Zhang, Qinglian Wen, Dan Li

**Affiliations:** ^1^ Department of Oncology, Affiliated Hospital of Southwest Medical University, Luzhou, Sichuan, China; ^2^ Department of Radiation Oncology, Cancer Center, West China Hospital, Sichuan University, Chengdu, Sichuan, China

**Keywords:** multiple intracranial brain metastases, cervical small cell carcinoma, whole-brain radiotherapy, sequential integrated boost radiation therapy, immunotherapy, vascular-targeted therapy

## Abstract

**Background:**

Cervical small cell carcinoma (CSCC) is a rare and highly aggressive malignancy with a poor prognosis. Brain metastases develop in 10–20% of patients, complicating clinical management and underscoring the need for effective therapeutic strategies.

**Case presentation:**

A 54-year-old female with CSCC developed 16 isolated brain metastases during treatment. She responded markedly to whole-brain radiotherapy (WBRT) combined with sequential integrated boost radiotherapy (SEB). Although recurrence emerged outside the SEB field one year later, subsequent treatment with immune checkpoint inhibitors and antiangiogenic agents induced complete remission (CR), achieving a progression-free survival (PFS) of 16 months. Remarkably, the patient has achieved an overall survival of 3 years since the diagnosis of brain metastases, without significant treatment-related cognitive impairment, and remains in CR.

**Conclusion:**

The combination of WBRT and SEB improves metastatic dose coverage in CSCC patients with multiple brain metastases. Furthermore, combining immunotherapy with antiangiogenic therapy demonstrates significant efficacy against post-radiation intracranial recurrence, supporting a multimodal individualized approach for further study.

## 1 Introduction

Cervical small cell carcinoma (CSCC) is an infrequent yet highly malignant subtype of cervical cancer, accounting for approximately 0.5-2% of all cases (1, 2). Morphologically, CSCC shares similarities with other small cell carcinomas, characterized by diminutive, round or spindle-shaped cells that exhibit diffuse and homogeneous distribution. This aggressive malignancy demonstrates a strong propensity for metastasis and recurrence (1, 3, 4). Despite treatment guidelines primarily drawing from those established for Small cell lung cancer (SCLC) and conventional cervical tumors, the prognosis of CSCC remains notably inferior to that of the typical cervical cancer. The five-year survival rate for early-stage CSCC is reported to be approximately 30%, which significantly decreases to around 10% among patients with advanced disease (4–6).

Metastasis frequently occurs in CSCC, particularly involving lymph nodes at an early stage, as well as distant sites such as the lungs, liver, bones, and brain. Reportedly, the occurrence rate of brain metastasis during the progression of CSCC ranges from 10% to 20% (7, 8), and it is notably linked to an unfavorable prognosis. The median survival time from the diagnosis of brain metastasis to mortality is recorded as 6.2 months (range: 0.1-21.1 months) (9). This discovery has garnered substantial attention within the academic and clinical domains. While prophylactic whole-brain radiotherapy (WBRT) has demonstrated efficacy in prolonging survival and alleviating symptoms associated with brain metastasis in small cell lung cancer (SCLC), there is limited evidence from large-scale randomized controlled trials supporting its use in CSCC (10). Therefore, the prophylactic application of WBRT in CSCC remains a subject of ongoing debate, despite it being a valuable treatment modality for brain metastasis. Furthermore, although radiation therapy often achieves effective reduction of primary lesions, there may be limitations in dosage that result in intracranial lesion progression within a few months for certain patients. Determining appropriate treatment strategies for these patients to improve their survival rates and quality of life presents a challenging inquiry in contemporary clinical research.

In this article, we present a case study of a patient diagnosed with CSCC who received treatment at our institution. Following chemoradiotherapy, the patient did not receive prophylactic WBRT and subsequently developed intracranial metastasis within six months. In response to this clinical scenario, salvage WBRT was administered along with SEB therapy and combination chemotherapy, resulting in complete remission (CR) that was sustained for 1 year. After further only intracranial progression, the patient underwent combination therapy involving immunotherapy and targeted therapy, achieving another CR with ongoing progression-free survival (PFS) lasting for 12 months. This case study provides valuable insights and guidance for the diagnosis and treatment of similar patients in clinical practice.

## 2 Case presentation

In February 2021, a 50-year-old woman sought medical consultation due to a chief complaint of irregular vaginal bleeding that had persisted for over three months. The patient is in overall good health, with no notable medical history or documented familial predisposition to tumors. Following a comprehensive diagnostic evaluation, she was subsequently diagnosed with stage IIIC1 cervical small cell carcinoma. The immunohistochemical analysis revealed the following: CK7 (-), CK20 (-), CEA (-), Vim (-), P16 (+), P53 (+, 1%), Ki67 (+, 80%), CD56 (-), CgA (+), Syn (+), CK (partially +). From March 16, 2021, to August 12, 2021, the patient underwent Six cycles of chemotherapy utilizing the EP regimen (Etoposide 100mg/m^2^, ivgtt, d1-3 + Cisplatin 25mg/m^2^, ivgtt, d1-3, Q3w). Concurrently, she underwent radical intensity-modulated radiotherapy (IMRT, PTV 45 Gy/25 F/5 W, PGTVnd 60 Gy/25 F/5 W) on April 9, 2021. Subsequently, CT-guided 3D-conformal brachytherapy (7 Gy × 4 F) was administered starting May 28, 2021, achieving cumulative EQD2 (α/β = 10) D90 doses of 92.1 Gy for the high-risk clinical target volume (HR-CTV) and 70.7 Gy for the intermediate-risk clinical target volume (IR-CTV), consistent with GEC-ESTRO guidelines. All organ-at-risk (OAR) dose constraints were maintained within established tolerance limits. In September 2021, the patient’s condition was determined to have achieved complete remission (CR) based on imaging evaluation. Subsequently, regular follow-up examinations were conducted.

**Figure 1:**
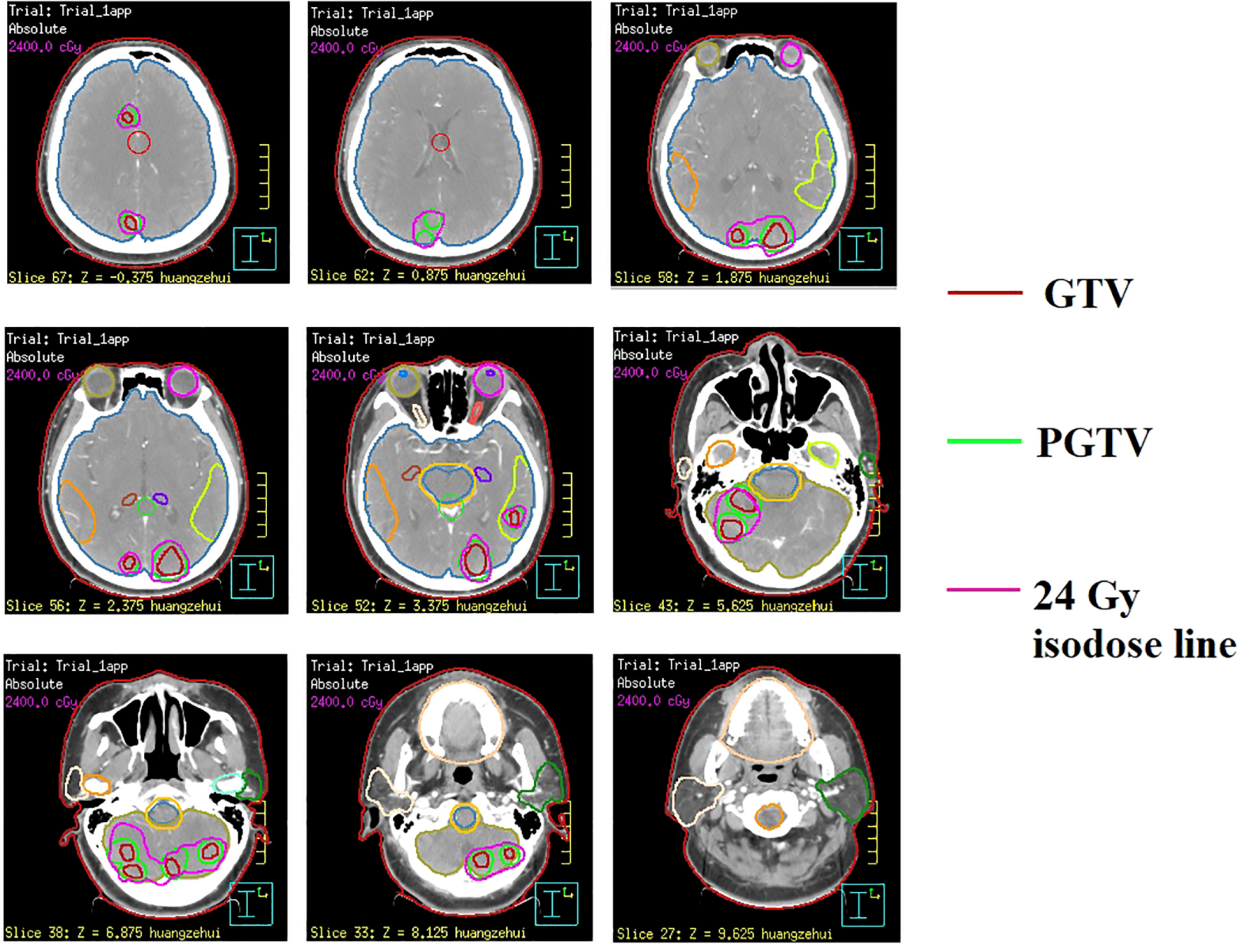
Illustration of radiotherapy target volumes in sequential integrated boost radiation therapy (SEB) at representative CT levels.

In February 2022, the patient presented with mild dizziness and unsteady gait. Head magnetic resonance imaging (MRI) revealed multiple brain metastases in the bilateral frontal, parietal, temporal, and occipital lobes, as well as in the cerebellar hemisphere, without evidence of extracranial tumor recurrence. Based on these clinical findings, the patient received WBRT on February 22, 2022, utilizing a Varian Clinac 6EX linear accelerator with 6MV photon beams. Radiation treatment was administered using a single-isocenter technique with coplanar, parallel-opposed lateral beams to ensure comprehensive target coverage (PTV 30 Gy/10 F/2 W). Additionally, sequential integrated boost radiation therapy (SEB) was administered on March 10, 2022 (IMRT, PTV 24 Gy/12 F/3 W). The cumulative equivalent dose in 2 Gy fractions (EQD2; α/β = 3) to organs at risk (OARs) was quantified as follows: brainstem (51.2 Gy), optic chiasm (left: 40.9 Gy; right: 41.3 Gy), lens (left: 5.3 Gy; right: 5.2 Gy), and globe (left: 4.18 Gy; right: 3.6 Gy). All dose parameters remained within established clinical tolerance limits. Owing to the extensive intracranial metastatic burden and the necessity for comprehensive target coverage, strict adherence to RTOG 0933 hippocampal dose constraints was not feasible. Quantitative dosimetry demonstrated mean/maximum hippocampal doses of 3.8 Gy/43.8 Gy (left) and 3.9 Gy/45.5 Gy (right). Following multidisciplinary risk-benefit evaluation, we implemented a clinically justified dose optimization strategy that prioritized tumor control while minimizing neurocognitive risks. Refer to **Figure 1** for the diagram of the radiotherapy target area. Following this treatment regimen, the patient completed four cycles of adjuvant chemotherapy (cyclophosphamide 750 mg/m^2^, ivgtt, d1 + doxorubicin hydrochloride 50 mg/m^2^, ivgtt, d1 + vincristine 1.4 mg/m^2^, iv, d1, Q3w). Subsequently, the treatment strategy was adjusted to maintenance therapy using capecitabine (2 g, po, Bid) after achieved partial remission (PR) based on imaging evaluation, as the patient exhibited intolerance towards chemotherapy.

**Figure 2:**
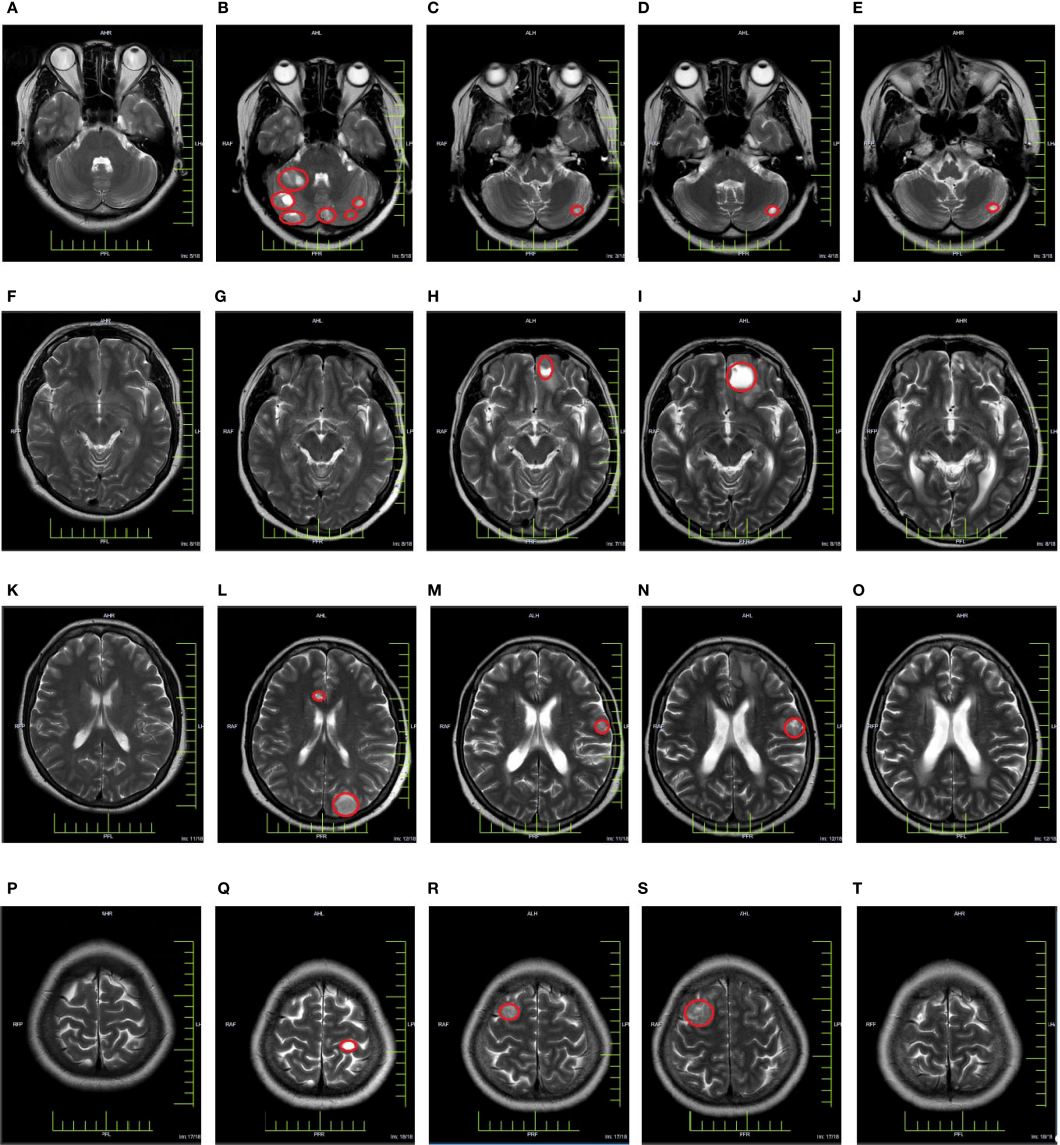
Cranial MRI evaluation map [**(A, F, K, P)** In March 2021, a baseline cranial MRI evaluation was performed, revealing no apparent abnormalities in the cranium. **(B, G, L, Q)** In March 2022, a follow-up cranial MRI examination was conducted, revealing the presence of multiple metastatic lesions. **(C, H, M, R)** In March 2023, a follow-up cranial MRI examination was conducted, revealing the presence of newly detected lesions in the cranium **(D, I, N, S)** In October 2023, a follow-up cranial MRI examination was performed, revealing an increasing trend in the size of the lesion. **(E, J, O, T)** In December 2023, a follow-up cranial MRI examination was conducted, revealing a significant reduction or disappearance of metastatic lesions].

In March 2023, a subsequent MRI examination unveiled the emergence of newly developed intracranial metastases in the patient. Despite being offered immune combined system chemotherapy, the patient declined and expressed a preference to continue with oral chemotherapy medication. In October 2023, the patient experienced worsening symptoms of dizziness and headache. Subsequent examination revealed an enlargement of lesions in bilateral frontal and temporal lobes, while no progression was observed in other sites throughout the body. After extensive communication with the patient, she remained steadfast in her refusal of immunotherapy combined with systemic chemotherapy. However, she expressed willingness to undergo immunotherapy coupled with targeted therapy. Taking into account economic factors, the patient opted for off-label use of sintilimab and signed an informed consent. Consequently, her treatment regimen was formulated as a combination therapy involving sintilimab and bevacizumab. (bevacizumab 15 mg/kg, d1, ivgtt + sintilimab 200 mg, d1, Q3W). Encouragingly, CR was achieved following the assessment of 4 treatment cycles. Currently, the maintenance treatment has been maintained for a period of 16 months. Important imaging evaluations of this patient over 3 years are shown in **Figure 2**.

## 3 Discussion

**Figure 3:**
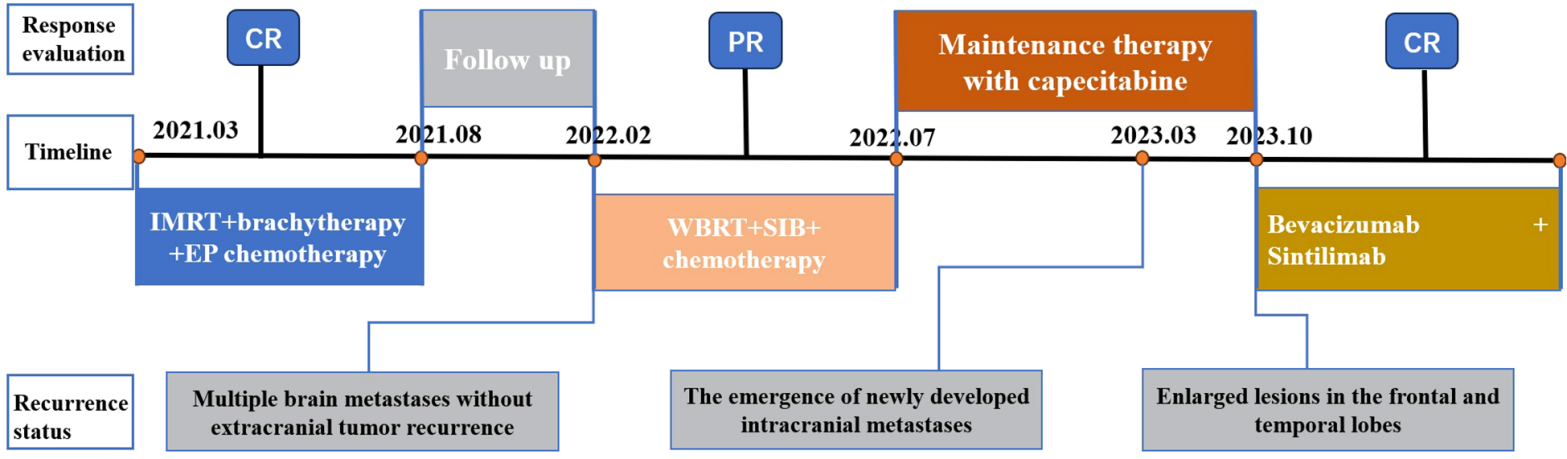
Treatment flow chart.

CSCC is an infrequent yet highly aggressive malignancy that was initially documented by Albores-Saavedra et al. in 1972 (11). The diagnostic and therapeutic strategies for CSCC have been primarily drawn from the methodologies employed in addressing SCLC and prevalent cervical tumors. However, due to its inherent predisposition for distant metastasis and rapid relapse even after achieving complete remission, each treatment modality encounters substantial challenges when applied to CSCC patients. Here, we present a case report of a patient diagnosed with stage III C1 CSCC three years ago. Encouragingly, the patient has reached an important survival milestone of 30 months since the occurrence of brain metastasis and is presently in CR stage. The comprehensive treatment course is illustrated in **Figure 3**. Throughout the entire treatment process, we have identified three key issues worthy of discussion and critical insights for further analysis and experience accumulation.

The first key question concerns whether prophylactic WBRT should be considered following primary treatment with combined radiotherapy and EP chemotherapy. Notably, prophylactic WBRT was not administered. The incidence of brain metastasis in CSCC patients has been reported to range from 10% to 20%, prompting the question of whether prophylactic WBRT is warranted. Limited statistics indicate that approximately 10% to 14% of patients with SCLC present with brain metastasis at diagnosis, while about 50% of SCLC patients develop brain metastasis within 2 years of diagnosis (12). Although the NCCN guidelines recommend prophylactic WBRT for SCLC, clinical trials and relevant guidelines supporting its use in CSCC are lacking. Controversy exists regarding whether the approach of prophylactic WBRT should be applied to CSCC similar to that of SCLC. Based on statistical analysis of extrapulmonary small cell carcinoma data, Naidoo et al. proposed that fundamental differences in biology and metastatic behavior do not warrant the routine use of prophylactic WBRT in CSCC (10). However, an analysis by Wang’s team suggests that while the risk of brain metastasis is relatively high in CSCC compared to other extrapulmonary sites, future studies may shed light on the suitability of prophylactic WBRT in CSCC treatment (7). Consequently, the debate regarding prophylactic WBRT for CSCC remains unresolved due to insufficient evidence. In this specific case, prophylactic WBRT was not administered after comprehensive communication with the patient.

Following six months of radical radiotherapy combined with EP systemic therapy, the patient presented with multiple metastatic lesions in the bilateral frontal, parietal, temporal, occipital lobes, and cerebellar hemispheres (a total of 12 lesions). It has been observed that while WBRT can encompass a wide range of lesions and contribute to disease control, its efficacy is compromised by lower dosages. Several prospective studies have provided evidence suggesting that in cases with a limited number of brain metastases, combining WBRT with lesion-targeted radiation therapy can enhance local control rates and extend overall survival in patients (13–16). Lesion-targeted radiation therapies encompass SEB and simultaneous integrated boost (SIB) techniques. Research data indicates that for patients with brain metastases, the utilization of a WBRT + SEB regimen results in improved survival outcomes compared to WBRT + SIB, potentially minimizing the incidence of treatment-induced neurocognitive impairments (17). However, caution should be exercised when applying this treatment approach to cases with more than 10 focal lesions. The second key question revolves around whether SEB therapy is indicated for the patient in this case. Considering the patient’s and their family’s positive outlook and the relatively safe locations of the brain metastases, salvage WBRT in conjunction with SEB was chosen. Significantly, we observed favorable disease control over a duration of one year using this combination therapy and a relapse was observed in the non-SEB treatment area. Notably, standardized assessment using the Montreal Cognitive Assessment (MoCA, version 8.2, https://mocacognition.com/) demonstrated preserved cognitive function (score: 22; normal ≥26) meeting criteria for mild cognitive impairment (18-25 points) per established guidelines. These findings suggest that integrating WBRT and SEB holds promise for improving patient prognosis in cases with a high number of intracranial metastases exceeding 10 but relatively safe relative locations.

Patients with brain metastases from small cell carcinoma often face high recurrence rate, and the presence of small residual lesions not targeted by SEB can give rise to challenges after WBRT. The third key question involves determining the optimal treatment strategy for cases of isolated intracranial recurrence in patients who have previously undergone WBRT plus SEB therapy. The NCCN guidelines advise caution when considering repeat WBRT in patients who have received this treatment previously, as repeated administration may result in severe cerebral adverse effects and functional impairment, causing discomfort and burden to the patients. Moreover, traditional chemotherapy agents face obstacles in effectively penetrating brain metastatic lesions attributed to the protective blood-brain barrier. Therefore, exploring salvage treatment strategies capable of enhancing quality of life and extending survival time for patients afflicted with cervical small cell carcinoma brain metastases is importance. In recent years, immune checkpoint inhibitors (ICIs) have emerged as promising modalities for treating brain metastases. Subgroup analysis of Keynote-024 (18) revealed that Pembrolizumab (n=18) exhibited a longer median PFS compared to chemotherapy (n=10) in patients with brain metastases. Similarly, retrospective analyses have corroborated that the nivolumab–ipilimumab combination confers significant overall survival benefits compared with chemotherapy alone (19). Within the EMPOWER-Lung 1 trial, Cemiplimab demonstrated extended overall survival (OS) of 18.7 months and PFS of 10.4 months among the subgroup of patients with brain metastases, surpassing chemotherapy’s OS of 11.7 months and PFS of 5.3 months (20). Analysis of data from the IMMUNED trial demonstrated that combination therapy with Nivolumab and Ipilimumab resulted in significantly lower recurrence rates than Nivolumab monotherapy for patients with a history of brain metastases (21), underscoring the importance of combination therapy. Furthermore, studies have illustrated that anti-angiogenic drugs can upregulate PD-L1 expression on tumor cells, remodel the immune microenvironment, and enhance the clinical effectiveness of ICIs (22, 23). Notably, they can also alleviate brain edema in patients with brain metastases. After comprehensive discussions with the patient and their family, immunotherapy combined with systemic chemotherapy was rejected in favor of immunotherapy combined with targeted therapy. Encouragingly, PR was observed at the 2-cycle assessment, progressing to CR by the 4-cycle assessment. Presently, the patient has achieved a PFS of 16 months, which is an encouraging outcome for a patient experiencing recurrent brain metastases after radiotherapy.

## 4 Conclusion

In the setting of CSCC with brain metastasis, the implementation of SEB therapy can address the issue of inadequate dosing to brain metastatic lesions observed with WBRT. Notably, the synergistic combination of immunotherapy and vascular-targeted drugs has demonstrated significant therapeutic efficacy in addressing recurrent brain metastasis in CSCC following radiotherapy. These findings provide compelling evidence for the potential adoption of this approach as an innovative treatment strategy for CSCC brain metastasis.

## 5 Limitations

The single-case report design precludes broad generalization of the results, necessitating future validation in large-scale randomized controlled trials. Furthermore, the absence of comprehensive biomarker profiling—including PD-L1 expression, microsatellite instability (MSI), and tumor mutational burden (TMB)—limits mechanistic insights into the observed effects.

## Data Availability

The raw data supporting the conclusions of this article will be made available by the authors, without undue reservation.
